# Acute Valve Syndrome in Aortic Stenosis

**DOI:** 10.1016/j.shj.2024.100377

**Published:** 2024-10-28

**Authors:** Philippe Généreux, Patricia A. Pellikka, Brian R. Lindman, Philippe Pibarot, Santiago Garcia, Konstantinos P. Koulogiannis, Evelio Rodriguez, Vinod H. Thourani, Michael Dobbles, Gennaro Giustino, Rahul P. Sharma, David J. Cohen, Allan Schwartz, Martin B. Leon, Linda D. Gillam

**Affiliations:** a Department of Cardiology, Gagnon Cardiovascular Institute, Morristown Medical Center, Morristown, New Jersey, USA; b Department of Cardiovascular Medicine, Mayo Clinic, Rochester, Minnesota, USA; c Division of Cardiovascular Medicine, Structural Heart and Valve Center, Vanderbilt University Medical Center, Nashville, Tennessee, USA; d Department of Cardiology Research, Quebec Heart & Lung Institute, Laval University, Quebec City, Quebec, Canada; e The Carl and Edyth Lindner Center for Research and Education, The Christ Hospital Physicians – Heart and Vascular, Cincinnati, Ohio, USA; f Department of Cardiothoracic Surgery, Ascension Saint Thomas, Nashville, Tennessee, USA; g Department of Cardiothoracic Surgery, Piedmont Heart and Vascular Institute, Atlanta, Georgia, USA; h egnite, Inc., Aliso Viejo, California, USA; i Department of Cardiovascular Medicine, Stanford University School of Medicine, Stanford, California, USA; j Clinical Trials Center, Cardiovascular Research Foundation, New York, New York, USA; k St. Francis Hospital and Heart Center, Roslyn, New York, USA; l Division of Cardiology, Columbia University Irving Medical Center, New York, New York, USA

**Keywords:** Aortic valve, Aortic stenosis, Aortic valve replacement, Clinical presentation, Transcatheter aortic valve replacement, Transcatheter aortic valve implantation

## Abstract

**Background:**

To describe the impact of clinical presentation among patients with aortic stenosis (AS) undergoing aortic valve replacement (AVR).

**Methods:**

We analyzed a real-world dataset including patients from 29 US hospitals (egnite Database, egnite). Patients over 18 years old with moderate or greater AS undergoing AVR were included. Patients were classified into 3 groups according to the acuity and severity of clinical presentation prior to AVR: (i) asymptomatic, (ii) progressive signs and symptoms (progressive valve syndrome [PVS]), and (iii) acute or advanced signs and symptoms (acute valve syndrome [AVS]). Mortality and heart failure hospitalization after AVR were examined with Kaplan-Meier estimates, with results compared using the log-rank test.

**Results:**

Among 2,009,607 patients in our database, 17,838 underwent AVR (78.6% transcatheter AVR, 21.4% surgical AVR). Age was 76.5 ± 9.7 years, and 40.2% were female. Prior to AVR, 2504 (14.0%) were asymptomatic, 6116 (34.3%) presented with PVS, and 9218 (51.7%) presented with AVS. At 2 years, the estimated rate of mortality for asymptomatic, PVS, and AVS were 5.8% (4.6%-7.0%), 7.6% (6.7%-8.4%), and 17.5% (16.5%-18.5%), respectively, and the estimated rate of hospitalization with heart failure for asymptomatic, PVS, and AVS were 11.1% (9.5%-12.6%), 19.0% (17.8%-20.2%), and 41.5% (40.2%-42.8%), respectively. After adjustment, patients presenting with AVS had increased risk of mortality after AVR (hazard ratio, 2.2; 95% CI, 1.8-2.6).

**Conclusions:**

From a large, real-world database of patients undergoing AVR for AS, most patients presented with AVS, which was associated with an increased risk of mortality and heart failure hospitalization.

## Highlights

• Seventeen thousand eight hundred thirty-eight patients with aortic stenosis underwent aortic valve replacement (AVR).
• Patients were classified into 3 groups according to their clinical presentation pre-AVR.
• Asymptomatic (14%), progressive valve syndrome (PVS; 34.3%), and acute valve syndrome (AVS; 51.7%).
• Two-year death post-AVR per presentation: asymptomatic 5.8%, PVS 7.6%, and AVS 17.5%.
• Two-year heart failure hospitalization: asymptomatic 11.1%, PVS 19.0%, and AVS 41.5%.

## Graphical abstract

Two-year mortality or hospitalization with heart failure after aortic valve replacement (AVR) for aortic stenosis per initial clinical presentation. From the egnite database, 17,838 patients with aortic stenosis underwent AVR. Patients were classified in 3 groups according to their clinical presentation pre-AVR. Before AVR, 14% were asymptomatic, 34.3% presented with progressive symptoms or progressive valve syndrome (PVS), and 51.7% presented with acute valve syndrome (AVS). Two-year mortality or hospitalization with heart failure post-AVR was the lowest in asymptomatic patients (14.9%) and increased with the severity and acuity of clinical presentation pre-AVR: PVS 23% and AVS 47.3%.

**Figure:**
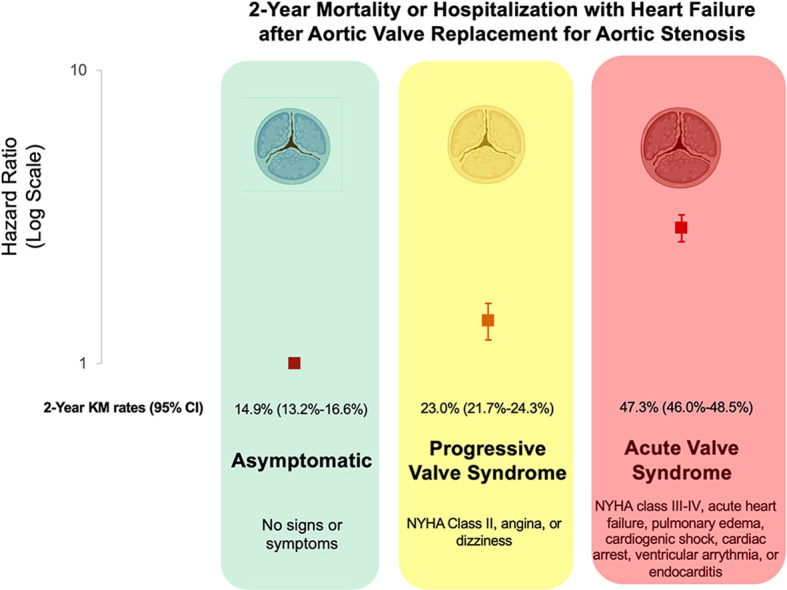

Aortic stenosis (AS) is a progressive disease characterized by periods of latency and variable evolution. Clinically, patients often present with no symptoms, especially in the early phase of the disease, and as valve degeneration and cardiac damage progress, a variety of symptoms and clinical manifestations occur. While some patients experience slow progression and develop mild symptoms such as fatigue and dyspnea, others present with a more unpredictable course, with more dramatic manifestations such as acute heart failure, syncope, shock, and even sudden death. Appropriate classification and characterization of these different clinical presentations is important, both for prognosis and therapeutic purposes. In the current manuscript, we explored the patterns of clinical presentation of AS among patients undergoing aortic valve replacement (AVR), and we propose a new classification related to the severity and acuity of the clinical presentation of AS: asymptomatic (no signs or symptoms), progressive signs or symptoms (progressive valve syndrome [PVS], and acute or advanced signs or symptoms (acute valve syndrome [AVS]). We evaluated the relationship of these classifications with outcome during the 2 years after AVR.

## Methods

The study population was drawn from 2,009,607 patients from 29 US institutions in the egnite Database (egnite) with appropriate permissions. Data were prepared for the present study following initial data quality assessments by a clinical team and evaluated for study inclusion and exclusion criteria. Key inclusion criteria included a documented diagnosis of moderate or greater AS per natural language processing (NLP)-based analysis of echocardiographic report(s), record of AVR procedure (which served as the study index date for purposes of this study), time from index date to censoring date >0 days, and ≥18 years of age at index. Patients were censored at date of last encounter within the health system or date of death. Key exclusion criteria included documented diagnosis of moderate or greater aortic regurgitation, AVR with missing date or prior to January 1, 2018 (for data quality purposes), and missing date of birth.

The resulting population was stratified into 3 cohorts based on severity and acuity of clinical presentation prior to AVR: (i) asymptomatic, with no signs or symptoms, (ii) progressive signs or symptoms of AS (PVS), such as New York Heart Association (NYHA) class II (dyspnea, fatigue, angina, or dizziness), diagnosed diastolic heart failure, edema, or elevated B-type natriuretic peptide (BNP) (100 < BNP < 400 pg/mL and/or 1000 pg/mL < N-terminal pro-B-type natriuretic peptide [NT-proBNP] < 1500 pg/mL), and (iii) acute or advanced signs and symptoms of AS (AVS), such as NYHA classes III-IV (any of hospitalization with heart failure, pulmonary edema, and syncope), left ventricle ejection fraction <50%, systolic heart failure, elevated BNP (BNP ≥400 pg/mL and/or NT-proBNP ≥1500 pg/mL), new-onset atrial fibrillation, hypotension, cardiogenic shock, new-onset ventricular arrhythmia, endocarditis, and resuscitation from cardiac arrest.

Patients were classified in one group based on the presence of ≥1 of the higher specified signs or symptoms, irrespective of the presence of other lesser signs or symptoms from the lower severity group. Patients were allocated to a specific cohort according to a 6-month lookback from the day before the index AVR.

Patients with AS were also classified according to AS severity (moderate, moderate-to-severe, and severe) per the documented diagnosis of AS in echocardiographic reports generated in the context of usual clinical practice. All available echocardiograms documented for a given patient were used to grade AS severity, and in the event of more than one available diagnosis, the date of the report with the first most severe documented diagnosis was used. Information on prespecified patient characteristics of interest was extracted as defined in Supplemental Table 1, with symptoms, acuity/severity of clinical presentation, comorbidity history, and treatment events identified according to relevant International Classification of Diseases, 10th Revision (ICD-10) codes, lab values, and/or echocardiographic report data documented in the 6 months lookback from index AVR, with definitions adjudicated by a clinical reviewer as well as a medical coding expert where relevant. Both the presence and severity of valve disease were derived from echocardiographic reports using a clinically reviewed and verified NLP algorithm (via random deidentified sampling exercise of 8000 echocardiographic reports) with an overall accuracy of >99% (Supplemental Table 2). NYHA II was defined by the presence of any of the following ICD-10 codes pre-AVR: dyspnea, dizziness, angina, and fatigue. NYHA III-IV was defined by the presence of any of the following ICD-10 codes pre-AVR: admission with heart failure, pulmonary edema, or syncope.

Two-year outcomes were evaluated using Kaplan-Meier (KM) estimates. The primary endpoint was all-cause mortality, where patients were censored at last clinical encounter. Information on patient death was extracted from medical records and reflects health care system/sites understanding of patient’s mortality. Secondary end points were hospitalization with heart failure and the composite of all-cause mortality or hospitalization with heart failure.

### Statistical Analysis

Patient characteristics were reported either as n (%) for categorical variables or as mean (SD) or median (interquartile range) for continuous variables as appropriate. KM estimates for all-cause mortality, hospitalization with heart failure, and the composite of all-cause mortality or hospitalization with heart failure post-AVR were reported per clinical presentation groups. Multivariable hazards analysis (Cox proportional hazards regression) was performed to simultaneously assess risk factors of interest for mortality, hospitalization with heart failure, and the composite of mortality or hospitalization with heart failure. A complete sensitivity analysis was also performed in which patients were allocated to cohorts according to a 12-month lookback from index AVR for presentation characteristics of interest. Unless otherwise stated, a p-value <0.05 was considered statistically significant, but with Bonferroni corrections for multiple comparisons. Analyses were completed using the following or greater: DataBricks 13.3 LTS; Apache Spark 3.4.1; Scala 2.12; R version 4.2.2; R survival package 3.5.3.

## Results

### Study Population

**Figure 1:**
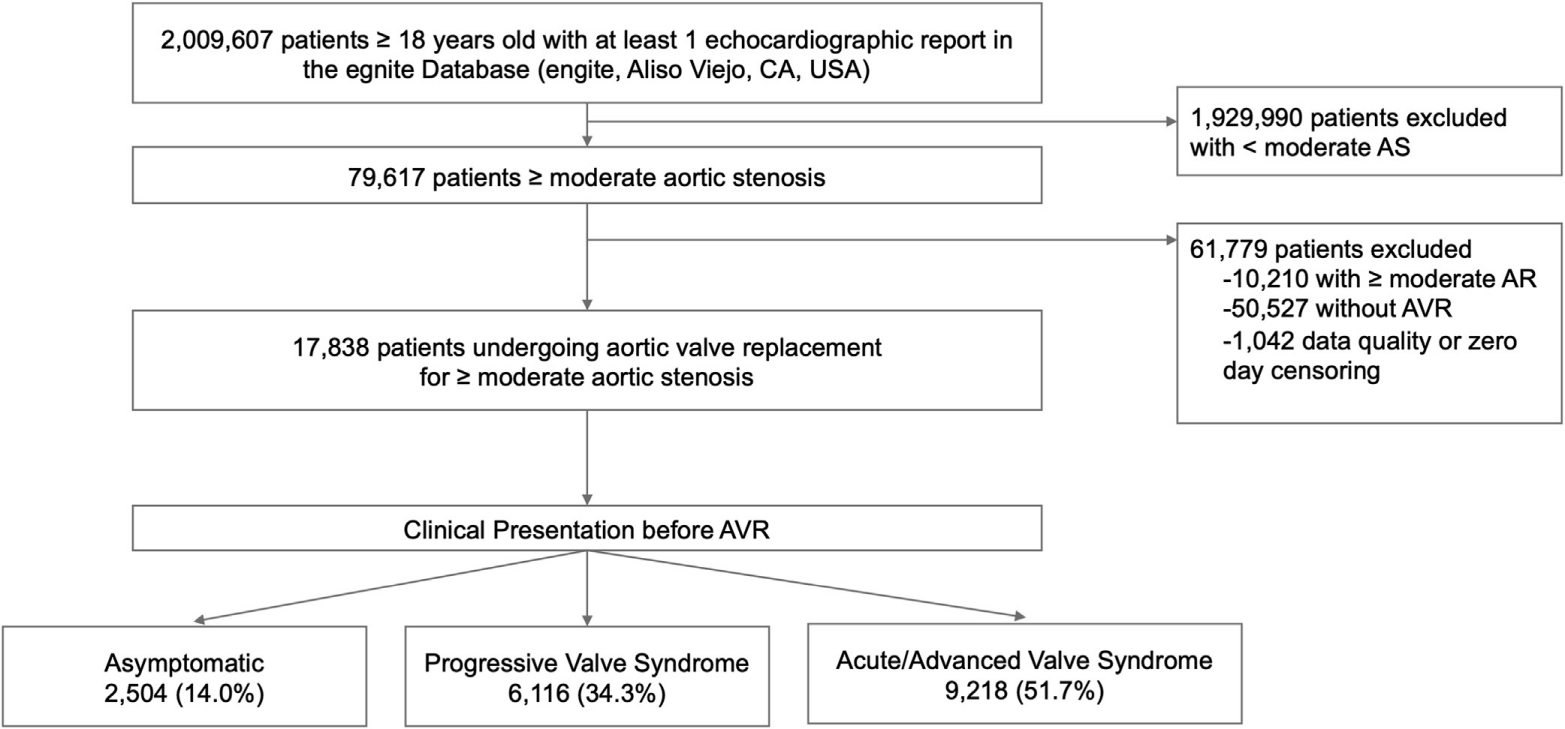
Study flow chart. Among 2,009,607 patients from 29 US institutions in the egnite Database (egnite), 17,838 patients with at least moderate aortic stenosis (AS) and undergoing aortic valve replacement (AVR) were included in our study. Patients were stratified in 3 groups based on the worst clinical presentation within 6 months prior to AVR: (i) asymptomatic, with no signs or symptoms, (ii) progressive signs or symptoms of AS (PVS), including: New York Heart Association (NYHA) class II (dyspnea, fatigue, dizziness, angina) edema, diastolic heart failure, and elevated natriuretic peptide defined as 100 pg/mL < BNP <400 pg/mL and/or 1000 pg/mL < NT-proBNP <1500 pg/mL, (iii) acute or advanced signs and symptoms of AS (AVS), including: NYHA class III-IV (any of admission with heart failure, pulmonary edema, or syncope), left ventricle ejection fraction <50% by echocardiogram, systolic heart failure, high natriuretic peptide defined as BNP ≥400 pg/mL and/or NT-proBNP ≥1500 pg/mL, new-onset atrial fibrillation, hypotension, cardiogenic shock, new-onset ventricular arrhythmia, resuscitation from cardiac arrest, or endocarditis. Abbreviations: AR, aortic regurgitation; AVS, acute valve syndrome; BNP, B-type natriuretic peptide; NT-proBNP, N-terminal pro-B-type natriuretic peptide; PVS, progressive valve syndrome.

Among a total of 2,009,607 patients with available echocardiographic reports and available clinical information, 17,838 underwent AVR (78.6% transcatheter, 21.4% surgical) for ≥moderate AS (Figure 1). Mean age was 76.5 ± 9.7 years, and 40.2% were female. At time of AVR, 2504 (14.0%) were asymptomatic, 6116 (34.3%) had PVS, and 9218 (51.7%) presented with AVS. The detailed lists of signs and symptoms included under each mode of presentation are shown in Table 1. Baseline characteristics per mode of clinical presentation are shown in Table 2. In general, patients presenting with AVS before AVR were older, more often male, had more comorbidities such as diabetes, coronary artery disease, and prior percutaneous revascularization, and presented more often with concomitant valve disease such as moderate or greater mitral or tricuspid valve regurgitation compared with asymptomatic patients or patients with PVS. Patients with AVS also had lower aortic valve peak velocity, lower mean gradient, and lower left ventricle ejection fraction before AVR.

**Table 1:** Clinical presentation before aortic valve replacement

Clinical presentation	N = 17,838
No signs or symptoms	2504 (14.0%)
Progressive valve syndrome (PVS)	6116 (34.3%)
New York Heart Association class II∗	4575 (74.8%)
Dyspnea	3999 (65.4%)
Diastolic heart failure	2997 (49%)
Fatigue	870 (14.2%)
Edema	630 (10.3%)
Elevated natriuretic peptide†	529 (8.6%)
Dizziness	488 (8%)
Angina	446 (7.3%)
Acute or advanced valve syndrome (AVS)	9218 (51.7%)
New York Heart Association class III-IV‡	5918 (64.6%)
Admission with heart failure	4651 (50.5%)
Left ventricle ejection fraction <50% (echocardiography)	3307 (35.9%)
Systolic heart failure	3212 (34.8%)
High natriuretic peptide§	2227 (24.2%)
New-onset atrial fibrillation	1943 (21.1%)
Syncope	1494 (16.2%)
Hypotension	1295 (14%)
Pulmonary edema	1049 (11.4%)
Cardiogenic shock	357 (3.9%)
New-onset ventricular arrhythmia	329 (3.6%)
Endocarditis	76 (0.8%)
Resuscitation from cardiac arrest	39 (0.4%)

*Notes.*Values are n (%).

Abbreviations: BNP, B-type natriuretic peptide; NT-proBNP, N-terminal pro-B-type natriuretic peptide.

∗ Any of dyspnea, dizziness, angina, and fatigue.

† Defined as 100 pg/mL < BNP < 400 pg/mL and/or 1000 pg/mL < NT-proBNP < 1500 pg/mL.

‡ Any of admission with heart failure, pulmonary edema, or syncope.

§ Defined as BNP ≥400 pg/mL and/or NT-proBNP ≥1500 pg/mL.

**Table 2:** Baseline characteristics of the study population

	Overall	Asymptomatic	PVS	AVS	*p* value
	17,838	2504	6116	9218	
Age, y	76.5 ± 9.7	74.0 ± 10.5	76.2 ± 9.0	77.4 ± 9.7	<0.001
Sex					<0.001
Female	7178 (40.2)	967 (38.6)	2625 (42.9)	3586 (38.9)	
Male	10,508 (58.9)	1503 (60.0)	3448 (56.4)	5557 (60.3)	
Unknown	152 (0.9)	34 (1.4)	43 (0.7)	75 (0.8)	
Body mass index, kg/m^2^	29.1 ± 8.5	29.3 ± 9.5	29.7 ± 8.2	28.6 ± 8.5	<0.001
Aortic valve area, cm^2^	0.8 ± 0.3	0.8 ± 0.2	0.8 ± 0.2	0.8 ± 0.3	<0.001
Peak velocity, m/s	4.0 ± 0.8	4.1 ± 0.7	4.1 ± 0.7	3.9 ± 0.8	<0.001
Mean gradient, mm Hg	39.6 ± 14.0	41.8 ± 13.4	40.8 ± 12.9	38.2 ± 14.7	<0.001
Left ventricle ejection, %	56.1 ± 12.0	61.1 ± 6.3	61.1 ± 6.3	50.6 ± 13.8	<0.001
Aortic stenosis severity					0.10
Moderate	1374 (7.7)	183 (7.3)	462 (7.6)	433 (7.9)	
Moderate to severe	1635 (9.2)	200 (8.0)	608 (9.9)	488 (8.9)	
Severe	14,829 (83.1)	2121 (84.7)	5046 (82.5)	4587 (83.3)	
Hypertension	14,272 (80.0)	1480 (59.1)	5156 (84.3)	7636 (82.8)	<0.001
Diabetes	6052 (33.9)	520 (20.8)	2018 (33.0)	3514 (38.1)	<0.001
Stroke	1018 (5.7)	79 (3.2)	310 (5.1)	629 (6.8)	<0.001
Coronary artery disease	12,868 (72.1)	1279 (51.1)	4558 (74.5)	7031 (76.3)	<0.001
Prior myocardial infarction	1650 (9.2)	84 (3.4)	200 (3.3)	1366 (14.8)	<0.001
Prior PCI	2068 (11.6)	179 (7.1)	550 (9.0)	1339 (14.5)	<0.001
Prior CABG	189 (1.1)	15 (0.6)	46 (0.8)	128 (1.4)	<0.001
COPD	2114 (11.9)	101 (4.0)	612 (10.0)	1401 (15.2)	<0.001
COPD on O_2_	319 (1.8)	6 (0.2)	39 (0.6)	274 (3.0)	<0.001
Chronic kidney disease	4411 (24.7)	219 (8.7)	1068 (17.5)	3124 (33.9)	<0.001
Moderate or greater MR	1964 (11.0)	113 (4.5)	349 (5.7)	1502 (16.3)	<0.001
Moderate or greater TR	1675 (9.4)	103 (4.1)	302 (4.9)	1270 (13.8)	<0.001
Transcatheter AVR	14,024 (78.6)	1666 (66.5)	4857 (79.4)	7501 (81.4)	<0.001
Surgical AVR	3814 (21.4)	838 (33.5)	1259 (20.6)	1717 (18.6)	<0.001
Concomitant PCI	233 (1.3)	16 (0.6)	70 (1.1)	147 (1.6)	<0.001
Concomitant CABG	1608 (9.0)	306 (12.2)	513 (8.4)	789 (8.6)	<0.001
Concomitant surgical MV repair/replacement	309 (1.7)	31 (1.2)	73 (1.2)	205 (2.2)	<0.001
Concomitant surgical TV repair/replacement	86 (0.5)	5 (0.2)	19 (0.3)	62 (0.7)	<0.001

*Notes.* Values are mean ± SD or n (%).

Abbreviations: AVR, aortic valve replacement; AVS, acute valve syndrome; CABG, coronary artery bypass grafting; COPD, chronic obstructive pulmonary disease; MR, mitral regurgitation; MV, mitral valve; PCI, percutaneous coronary intervention; PVS, progressive valve syndrome; TR, tricuspid regurgitation; TV, tricuspid valve.

### Mortality and Hospitalization for Heart Failure Per Clinical Presentation

**Figure 2:**
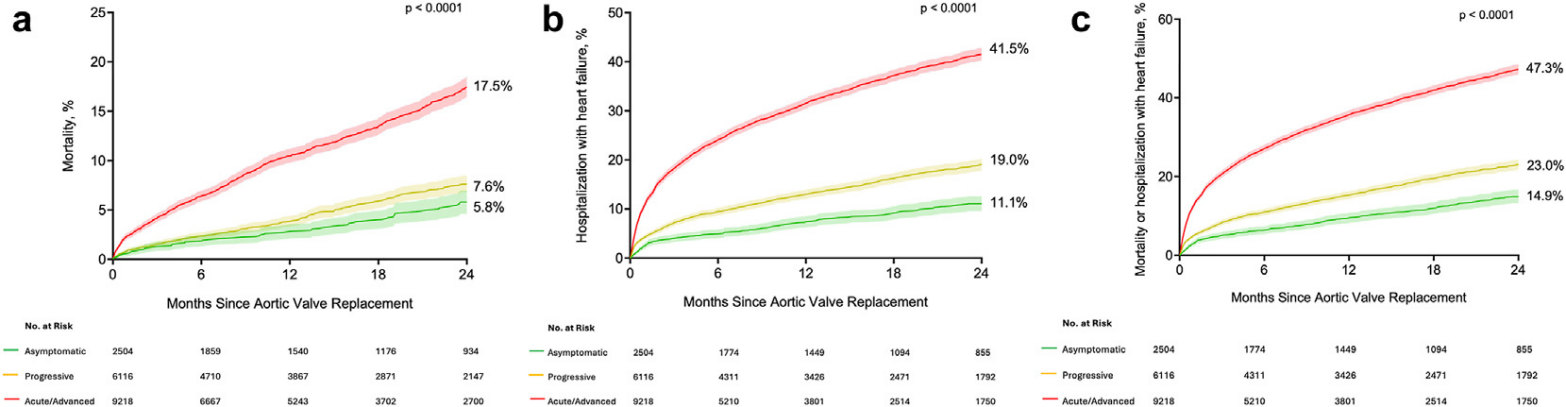
Two-year outcomes after aortic valve replacement (AVR) per clinical presentation. Two-year outcomes after AVR according to clinical presentation before AVR among patients with at least moderate aortic stenosis. (a) Two-year mortality after AVR per clinical presentation before AVR. (b) Two-year hospitalization with heart failure after AVR per clinical presentation before AVR. (c) Two-year mortality or hospitalization with heart failure after AVR per clinical presentation before AVR.

Two-year KM estimates for all-cause mortality after AVR were 5.8% (95% CI, 4.6%-7.0%) for asymptomatic patients, 7.6% (95% CI, 6.7%-8.4%) for patients presenting with PVS, and 17.5% (95% CI, 16.5%-18.5%) for patients presenting with AVS (Figure 2a; *p* < 0.0001). Two-year KM estimates for hospitalization with heart failure after AVR were 11.1% (95% CI, 9.5%-12.6%), 19.0% (95% CI, 17.8%-20.2%), and 41.5% (95% CI, 40.2%-42.8%), respectively (Figure 2b; *p* < 0.0001). Two-year KM estimates for the composite of all-cause mortality or hospitalization for heart failure are shown in Figure 2c (asymptomatic: 14.9%; 95% CI, 13.2%-16.6%; PVS: 23.0%; 95% CI, 21.7%-24.3%; and AVS: 47.3%; 95% CI, 46.0%-48.5%, *p* < 0.0001).

### Multivariable and Sensitivity Analysis

**Figure 3:**
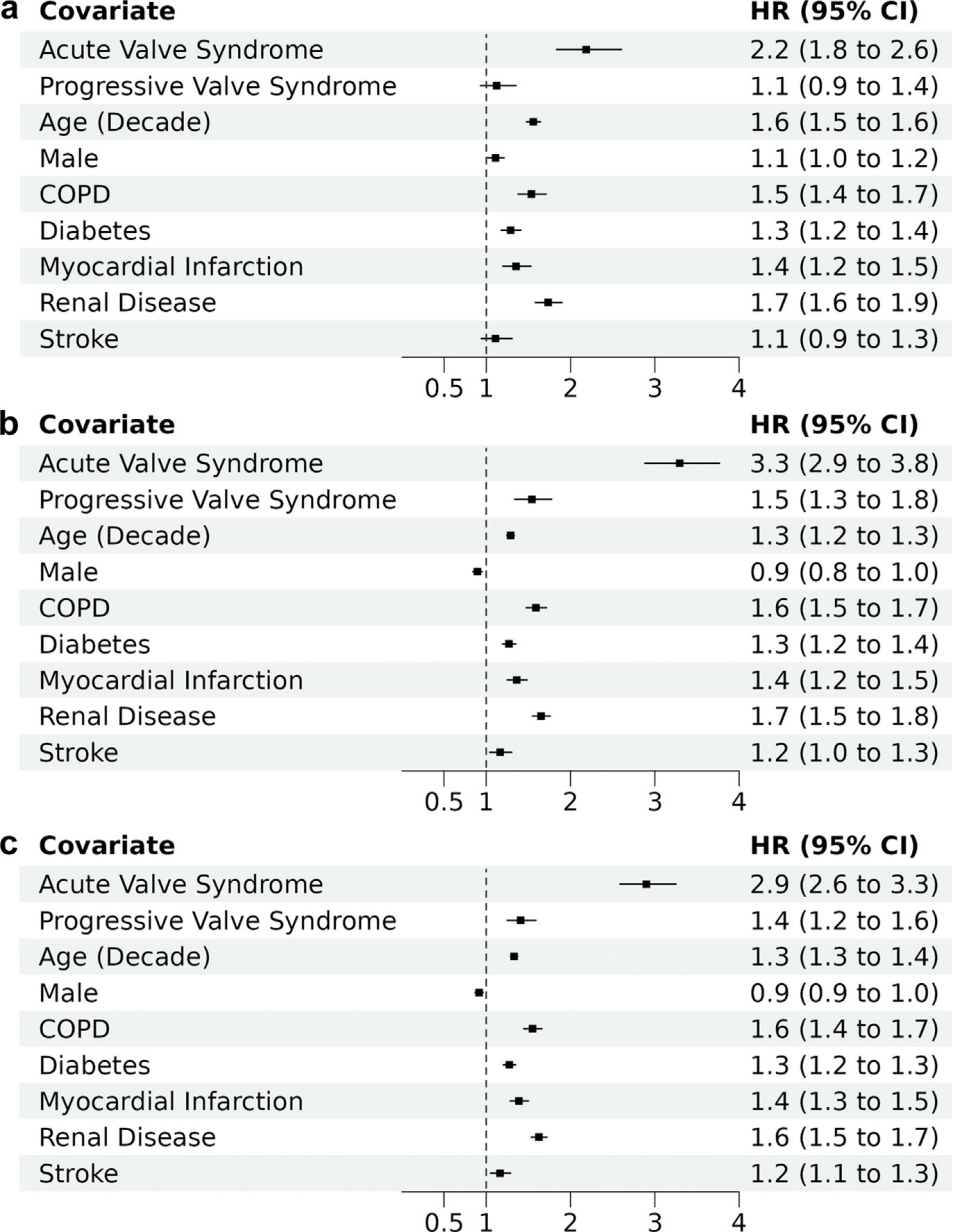
Multiple variable analysis for 2-year outcomes after aortic valve replacement. Two-year outcomes adjusted for age, sex, comorbidities, and clinical presentation before aortic valve replacement. (a) Mortality; (b) hospitalization for heart failure; (c) composite of mortality or hospitalization with heart failure. Abbreviation: COPD, chronic obstructive pulmonary disease; HR, hazard ratio.

Modeled hazards analysis demonstrated an incremental increase in all-cause mortality and heart failure hospitalization after AVR among patients presenting with more advanced signs or symptoms compared with asymptomatic presentation. After adjustment, compared to asymptomatic patients, those presenting with AVS had increased risk of mortality after AVR (adjusted hazard ratio [HR], 2.2; 95% CI, 1.8-2.6) (Figure 3a). Progressive valve syndrome and AVS were both independently associated with increased hazard of hospitalization with heart failure (PVS: adjusted HR, 1.5; 95% CI, 1.3-1.8; AVS: adjusted HR, 3.3; 95% CI, 2.9-3.8) (Figure 3b), and increased hazard of the composite of mortality or hospitalization with heart failure (PVS: adjusted HR, 1.4; 95% CI, 1.2-1.6; AVS: adjusted HR, 2.9; 95% CI, 2.6-3.3) (Figure 3c). Sensitivity analysis using 12-month lookback from AVR according to clinical presentation demonstrated similar results (Supplemental Figure 1 and 2). Supplemental Table 1 and Figure 3 show additional analyses stratifying patients in 4 groups (asymptomatic vs. progressive vs. advanced vs. acute) with similar findings.

## Discussion

The current study proposed an intuitive and novel clinical classification to better characterize signs and symptoms for patients presenting with AS. Derived from a large, real-world database, patients undergoing AVR for AS were classified into 3 categories based on the severity and acuity of AS signs and symptoms: asymptomatic presentation, PVS, and AVS. The main findings are as follows: (i) presentation with acute and advanced signs and symptoms was the most frequent mode of clinical presentation before AVR, with 51.7% of patients presenting with AVS; (ii) presentation with AVS was independently associated with a ∼3-fold increase in estimated rate of mortality and ∼4-fold increase in estimated rate of heart failure hospitalization at 2 years after AVR compared with asymptomatic patients; and (iii) PVS presentation was associated with a ∼40% increase in hospitalization with heart failure and ∼35% increase in the risk of mortality or heart failure hospitalization after AVR.

Our study demonstrated that more than half of patients undergoing AVR for moderate or severe AS in typical clinical settings presented with advanced or acute signs and symptoms. Our findings expand on prior studies, showing that up to ∼40% of patients present with acutely decompensated heart failure prior to AVR, with a significant increase in mortality, repeat heart failure hospitalization, and health care cost.^1^, ^2^, ^3^, ^4^, ^5^, ^6^, ^7^ Chen et al.^8^ showed that 40.7% of patients with severe symptomatic AS enrolled in the PARTNER 2 trial presented with acute heart failure hospitalization within 6 months prior to their AVR, with a ∼30% increase in mortality at 2 years compared with patients with no prior heart failure hospitalization. Similarly, Zilberszac et al.^9^ demonstrated that among patients followed regularly for severe AS with no symptoms by a dedicated heart valve clinic, 43% developed NYHA class III-IV heart failure symptoms when they first developed symptoms. The high proportions of patients presenting with AVS in our study could be explained by the real-world nature of our study, where undertreatment and delayed referral are frequent.^10^, ^11^, ^12^, ^13^

Patients undergoing AVR while being asymptomatic had lower rates of mortality and hospitalization for heart failure compared with patients presenting with PVS or AVS in our study. This finding illustrates the potential benefits of early intervention among patients with moderate or severe AS, both to prevent the progression of cardiac damage and to improve outcomes post-AVR.^14^^,^^15^ To date, 2 small randomized trials have demonstrated a benefit in surgical AVR among patients with asymptomatic, very severe AS^16^ and severe AS and normal low-level stress test.^17^ Patients included in those studies were in general younger, with a mean age of ∼65 years. Two additional randomized trials that included older patients treated with transcatheter AVR have currently completed enrollment and will be presented soon—the Evaluation of TAVR Compared to Surveillance for Patients With Asymptomatic Severe Aortic Stenosis (EARLY TAVR)^18^ and Early Valve Replacement Guided by Biomarkers of left ventricle Decompensation in Asymptomatic Patients With Severe AS (EVoLVeD).^19^ Those studies will help to determine whether pre-emptive AVR should be performed among patients with severe AS and no symptoms. Findings of these trials will be important, especially given the fact that the long-term implications of performing AVR earlier than currently indicated (severe AS with symptoms or with reduced ejection fraction) are currently unknown, especially in light of the finite durability of bioprosthetic valve.

Patients presenting with AVS were older and more often had comorbidities such as concomitant coronary artery disease and significant mitral or tricuspid regurgitation. While those factors could be seen as confounders associated with increased mortality after AVR, they are also associated with a state of decreased “myocardial resilience” and increased vulnerability to pressure overload, with patients more prone to present with severe and profound symptoms. In addition, they may reflect mitral and tricuspid regurgitation that are the results of and not coincidental to advanced AS. Those patients may derive benefit from earlier treatment while still in their asymptomatic clinical phase. Similarly, some patients may have initially presented with no or mild symptoms and, while waiting for treatment, progressed and developed more advanced symptoms.^10^^,^^20^, ^21^, ^22^, ^23^ Current global initiatives have been initiated to decrease morbidity and mortality of severe AS by targeting AVR procedure within <3 months from first emergence of a class 1 indication for intervention, typically symptoms.^24^ The proposed classification and current findings may help characterize patients’ clinical status at time of first assessment (asymptomatic vs. PVS vs. AVS) and help guide/prioritize their treatment, with the goal of treating patients while stable, avoiding waiting until they transitioned to a decompensated state. More importantly, patients with AS may have been diagnosed for the first time while presenting in AVS, raising concern about the burden of undiagnosed AS in the general population and potentially advocating for large screening initiatives of at-risk patients.^13^^,^^24^

### Limitations

This study has several limitations. First, signs and symptoms were not extracted prospectively but rather from chart reviews derived from problem lists, lab values, and NLP-based interpretation of echocardiographic procedure reports. That being said, this process benefited from the rigor and additional adjudication from site coding experts, which optimized signs and symptoms documentation compared with a single clinician. Second, AS severity was determined by sites, and raw echocardiograms were not reviewed independently. Third, while we showed a high proportion of patients presenting with advanced symptoms, our study does not account for patients with significant AS who died at home or in the hospital before undergoing AVR and only included patients who survived prior to AVR. Fourth, we most likely underestimated the rate of rehospitalization for heart failure since some patients may have received follow-up or care at institution not included in egnite database. Fifth, it is possible that information on patient deaths as extracted from medical records (i.e., as documented by each site) could be incomplete. Sixth, data regarding the time period exposed to moderate or severe AS, with or without symptoms, before referral and AVR, was not available. Finally, we classified patients based on the most severe mode of presentation within 6 months before AVR; however, AS is a dynamic disease influenced by multiple factors, and patients could present with symptoms that fluctuate through time. Detailed characterization of signs and symptoms progression/regression was beyond the scope of the current manuscript.

In conclusion, most patients undergoing AVR for AS presented with acute or advanced signs and symptoms, which was associated with a significant increase in mortality and hospitalization for heart failure after AVR. AVR among asymptomatic patients was associated with the lowest risk of events at 2 years, even lower than those undergoing AVR prompted by progressive signs or symptoms. Earlier identification of patients with significant AS and prompt intervention, even before symptoms develop, may optimize outcomes among patients with AS.

## Ethics Statement

Exemption from institutional review board review was obtained for this study from WCG IRB. All deidentified datasets used were compliant with the Health Insurance Portability and Accountability Act (HIPAA).

## Funding

The current study was supported by egnite, Inc, with an unrestricted grant from 10.13039/100006520Edwards Lifesciences.

## Disclosure Statement

P. Généreux is a consultant for 4C Medical, Abbott Vascular, Abiomed, BioTrace Medical, Boston Scientific, Caranx Medical, Cardiovascular Systems Inc, Edwards Lifesciences, Medtronic, Opsens, Pi-Cardia, Puzzle Medical, Shockwave, Soundbite Medical Inc, egnite, Inc, and Teleflex; is an advisor to Abbott Vascular, Abiomed, Edwards Lifesciences, egnite, Inc, and Medtronic; receives speaker fees from Abbott Vascular, Abiomed, Medtronic, Shockwave; is a principal investigator of 4C Medical for the AltaValve feasibility study, Cardiovascular Systems Inc/Abbott for the Eclipse Trial, and Edwards Lifesciences for the EARLY-TAVR and PROGRESS trials; has equity in Pi-Cardia, Puzzle Medical, and Soundbite Medical Inc; and proctor for and received institutional grants from Edwards Lifesciences. P. A. Pellikka received research support from Edwards Lifesciences (institution). B. Lindman has investigator research grants and consulting from Edwards Lifesciences and is a consultant at AstraZeneca. P. Pibarot has institutional funding from Edwards Lifesciences, Medtronic, Pi-Cardia, Cardiac Success, and Roche Diagnostics for echocardiography core laboratory analyses, blood biomarker analyses, and research studies in the field of interventional and pharmacologic treatment of valvular heart diseases, for which he received no personal compensation. S. Garcia is a steering committee member for Edwards (Alliance Trial) and Abbott Vascular (Envision Trial) and serves as a consultant and proctor for Medtronic, Abbott Vascular, and Edwards Lifesciences. K. P. Koulogiannis is a consultant at Edwards and Abbott; has core lab contracts with Abbott, Edwards Lifesciences, and Medtronic for which she receives no direct compensation; and has equity in 4C Medical. V. H. Thourani is on the advisory board and receives research support from Edwards Lifesciences, Artivion, Abbott Vascular, AtriCure, JenaValve Technology, Inc, Shockwave, Boston Scientific, Medtronic, and Dasi Simulations; is a member of advisory board and consultant for egnite, Inc. E. Rodriguez receives consulting fees, speaker fees, and honoraria from Edwards Lifesciences; receives consulting fees from egnite, Inc; receives consulting fees, speaker fees, clinical educator fees, and honoraria from AtriCure; receives consulting fees, speaker fees, honoraria, and a research grant from Abbott; receives speaker fees from Phillips; and receives consulting fees from CardioMech and Teleflex. R. P. Sharma receives consulting fees, speaker fees, and honoraria from Edwards Lifesciences; receives consulting fees and equity interest in egnite, Inc; and receives speaker fees and honoraria from Boston Scientific and Abbott. M. Dobbles has equity interest in and employee of egnite, Inc. D. J. Cohen receives research grant support from Edwards Lifesciences, Abbott, and Boston Scientific; receives consulting income from Edwards Lifesciences, Abbott, Boston Scientific, and Medtronic. M. Leon receives institutional clinical research support from Abbott, Boston Scientific, Edwards Lifesciences, Abiomed, and Medtronic. L. D. Gillam is a consultant for Edwards Lifesciences, Medtronic, and Philips; advisory board for egnite, Inc; and has core lab contracts with Abbott, Edwards Lifesciences, and Medtronic for which she receives no direct compensation. The other authors had no conflicts to declare.

## Review Statement

Full responsibility for the editorial process for this article was delegated to Guest Editor: Jonathan Tobis, MD.
