# Supplementary material for: Acute Valve Syndrome in Aortic Stenosis
Source: Struct Heart. 2024 Oct 28;9(4):100377. doi: 10.1016/j.shj.2024.100377 (PMC12047511; doi:10.1016/j.shj.2024.100377)

Supplemental Material

Acute Valve Syndrome in Aortic Stenosis

Philippe Généreux, MD; Patricia A. Pellikka, MD; Brian Lindman, MD; Philippe Pibarot, DVM, PhD; Santiago Garcia, MD; Konstantinos P. Koulogiannis, MD; Evelio Rodriguez, MD; Vinod H. Thourani, MD; Michael Dobbles, MS; Gennaro Giustino, MD; Rahul P. Sharma, MBBS; David J. Cohen, MD, MSc; Allan Schwartz, MD; Martin B. Leon, MD; Linda D. Gillam, MD, MPH

Supplemental Table 1. Variable Definitions

Parameter	Definition(s)
Admission with heart failure	In patient admission with associated heart failure ICD-10 code
Age	Extracted from EMR data (deidentified with ages 90 or greater truncated to a single category of ≥90 Per Safe Harbor Methodology)
Angina	'I20.0','I20.9','I20.8'
Aortic valve area	Value extracted from echocardiographic procedure report
Aortic valve peak velocity	Value extracted from echocardiographic procedure report
Aortic valve mean pressure gradient	Value extracted from echocardiographic procedure report
Atrial fibrillation	'I48.0', 'I48.1', 'I48.11', 'I48.19', 'I48.2', 'I48.20', 'I48.21', 'I48.91'
AR	Presence and severity extracted from echocardiographic procedure reports using validated NLP
AS	Presence and severity extracted from echocardiographic procedure reports using validated NLP
CAD	'I25.10', 'I25.110', 'I25.111', 'I25.118', 'I25.119', 'I25.2', 'I25.3', 'I25.41', 'I25.42', 'I25.5', 'I25.6', 'I25.700', 'I25.701', 'I25.708', 'I25.709', 'I25.710', 'I25.718', 'I25.719', 'I25.720', 'I25.721', 'I25.728', 'I25.729', 'I25.750', 'I25.758', 'I25.759', 'I25.790', 'I25.798', 'I25.799', 'I25.810', 'I25.811', 'I25.812', 'I25.82', 'I25.83', 'I25.84', 'I25.89', 'I25.9', 'I20.1', 'I20.2', 'I21.01', 'I21.02', 'I21.09', 'I21.11', 'I21.19', 'I21.21', 'I21.29', 'I21.3', 'I21.4', 'I21.9', 'I21.A1', 'I21.A9', 'I22.0', 'I22.1', 'I22.2', 'I22.9', 'I23.2', 'I23.6', 'I23.7', 'I23.8', 'I24.0', 'I24.1', 'I24.8', 'I24.9', 'Z98.61'
CABG	'Z95.1', 'Z95.5', 'Z95.828', '33510', '33511', '33512', '33513', '33517', '33518', '33519', '33521', '33522', '33530', '33533', '33534', '33535', '33536', '60210', '70210', '00210', '0210093', '0210099', '021009W', '02100A3', '02100A9', '02100AC', '02100AW', '02100Z8', '02100Z9', '02100ZC', '0211093', '021109W', '02110AW', '02110Z9', '0212093', '021209W', '021309W'
Cancer (metastatic)	'C77.0', 'C77.1', 'C77.2', 'C77.3', 'C77.4', 'C77.5', 'C77.8', 'C77.9', 'C78.00', 'C78.01', 'C78.02', 'C78.1', 'C78.2', 'C78.39', 'C78.4', 'C78.5', 'C78.6', 'C78.7', 'C78.80', 'C78.89', 'C79.00', 'C79.01', 'C79.02', 'C79.10', 'C79.11', 'C79.19', 'C79.2', 'C79.31', 'C79.32', 'C79.40', 'C79.49', 'C79.51', 'C79.52', 'C79.60', 'C79.61', 'C79.62', 'C79.70', 'C79.71', 'C79.72', 'C79.81', 'C79.82', 'C79.89', 'C79.9'
Cardiogenic shock	'R57.0'
COPD	'J44.0', 'J44.1', 'J44.9'
Dementia	'F01.50', 'F01.51', 'F01.511', 'F01.518', 'F01.53', 'F01.B0', 'G30.0', 'G30.1', 'G30.8', 'G30.9'

Documented death	Expired patient status/date of death from institution
Documented evaluation by Heart Team	Patient evaluation by Heart Team (per provider list issued by each site)
Diabetes	'E10.10', 'E10.11', 'E10.21', 'E10.22', 'E10.29', 'E10.311', 'E10.319', 'E10.3219', 'E10.329', 'E10.3293', 'E10.3299', 'E10.3393', 'E10.3513', 'E10.3519', 'E10.3553', 'E10.3593', 'E10.3599', 'E10.36', 'E10.39', 'E10.40', 'E10.41', 'E10.42', 'E10.43', 'E10.49', 'E10.51', 'E10.52', 'E10.59', 'E10.610', 'E10.618', 'E10.621', 'E10.622', 'E10.628', 'E10.641', 'E10.649', 'E10.65', 'E10.69', 'E10.8', 'E10.9', 'E11.00', 'E11.01', 'E11.10', 'E11.11', 'E11.21', 'E11.22', 'E11.29', 'E11.311', 'E11.319', 'E11.321', 'E11.3211', 'E11.3212', 'E11.3213', 'E11.3219', 'E11.329', 'E11.3291', 'E11.3292', 'E11.3293', 'E11.3299', 'E11.331', 'E11.3311', 'E11.3312', 'E11.3313', 'E11.3319', 'E11.339', 'E11.3391', 'E11.3392', 'E11.3393', 'E11.3399', 'E11.3411', 'E11.3412', 'E11.3413', 'E11.3419', 'E11.3491', 'E11.3493', 'E11.3499', 'E11.351', 'E11.3511', 'E11.3512', 'E11.3513', 'E11.3519', 'E11.3522', 'E11.3531', 'E11.3532', 'E11.3551', 'E11.3552', 'E11.3553', 'E11.3559', 'E11.359', 'E11.3591', 'E11.3592', 'E11.3593', 'E11.3599', 'E11.36', 'E11.39', 'E11.40', 'E11.41', 'E11.42', 'E11.43', 'E11.44', 'E11.49', 'E11.51', 'E11.52', 'E11.59', 'E11.610', 'E11.618', 'E11.620', 'E11.621', 'E11.622', 'E11.628', 'E11.638', 'E11.641', 'E11.649', 'E11.65', 'E11.69', 'E11.8', 'E11.9', 'E13.00', 'E13.10', 'E13.11', 'E13.21', 'E13.22', 'E13.29', 'E13.311', 'E13.319', 'E13.3299' 'E13.39', 'E13.40', 'E13.42', 'E13.43', 'E13.49', 'E13.51', 'E13.59', 'E13.621', 'E13.628', 'E13.641', 'E13.649', 'E13.65', 'E13.69', 'E13.8', 'E13.9'
Diastolic Heart Failure	'I50.3','I50.31','I50.33'
Dizziness	'R42'
Edema	'R60.0','R60.1','R60.9'
Elevated BNP	defined as lab value result of 100 pg/mL < BNP < 400 pg/mL and/or 1000 pg/mL < NT-proBNP <1500 pg/mL
Endocarditis	'I33'
Fatigue	'R53.8','R53.0'
High BNP	defined as lab value result of BNP ≥400 pg/mL and/or NT-proBNP ≥1500 pg/mL
Hypertension	'I10', 'I11.0', 'I11.9', 'I12.0', 'I12.9', 'I13.0', 'I13.10', 'I13.11', 'I13.2', 'I15.0', 'I15.1', 'I15.2', 'I15.8', 'I15.9'
Hypotension	'I95'
LVEF	Value extracted from echocardiographic procedure report
MI	'I21.01', 'I21.02', 'I21.09', 'I21.11', 'I21.19', 'I21.21', 'I21.29', 'I21.3', 'I21.4', 'I21.9', 'I21.A1', 'I21.A9', 'I22.0', 'I22.1', 'I22.2', 'I22.9', 'I23.2', 'I23.6', 'I23.7', 'I23.8'

MR	Presence and severity extracted from echocardiographic procedure reports using validated NLP
MS	Presence and severity extracted from echocardiographic procedure reports using validated NLP
New-onset atrial fibrillation	Defined as first recorded diagnosis of atrial fibrillation. The new-onset diagnosis code must correlate with an ECG order.
New-onset ventricular arrythmia	Defined as first recorded diagnosis of ventricular arrythmia. The new-onset diagnosis code must correlate with an ECG order.
Patient sex	Extracted from EMR data
Pulmonary edema	'J81'
PCI	'0270346', '027034Z', '027035Z', '027036Z', '027037Z', '02703DZ', '02703ZZ', '0271356', '027135Z', '027136Z', '027137Z', '02713ZZ', '027236Z', '027337Z', '02C03ZZ', '02C13ZZ', '92920', '92921', '92924', '92928', '92929', '92933', '92934', '92937', '92941', '92943', '92944', '92973', '92980', '92981', '92982', '92986', 'C9600', 'C9601', 'C9602', 'C9604', 'C9607'
Resuscitation from cardiac arrest	'M96.A'
Chronic kidney disease	'N18', 'N18.1', 'N18.2', 'N18.3', 'N18.30', 'N18.31', 'N18.32', 'N18.4', 'N18.5', 'N18.6', 'N18.9', 'N19', 'Z91.15', 'Z99.2', 'I12.0', 'I12.9', 'I13.0', 'I13.10', 'I13.11', 'I13.2'
Stroke	'I63.00', 'I63.011', 'I63.012', 'I63.013', 'I63.019', 'I63.02', 'I63.031', 'I63.032', 'I63.033', 'I63.039', 'I63.09', 'I63.10', 'I63.111', 'I63.112', 'I63.119', 'I63.12', 'I63.131', 'I63.132', 'I63.133', 'I63.139', 'I63.19', 'I63.20', 'I63.211', 'I63.212', 'I63.213', 'I63.219', 'I63.22', 'I63.231', 'I63.232', 'I63.233', 'I63.239', 'I63.29', 'I63.30', 'I63.311', 'I63.312', 'I63.313', 'I63.319', 'I63.321', 'I63.322', 'I63.329', 'I63.331', 'I63.332', 'I63.339', 'I63.341', 'I63.342', 'I63.39', 'I63.40', 'I63.411', 'I63.412', 'I63.413', 'I63.419', 'I63.421', 'I63.422', 'I63.423', 'I63.429', 'I63.431', 'I63.432', 'I63.433', 'I63.439', 'I63.441', 'I63.442', 'I63.443', 'I63.449', 'I63.49', 'I63.50', 'I63.511', 'I63.512', 'I63.513', 'I63.519', 'I63.521', 'I63.522', 'I63.523', 'I63.529', 'I63.531', 'I63.532', 'I63.533', 'I63.539', 'I63.541', 'I63.542', 'I63.543', 'I63.549', 'I63.59', 'I63.6', 'I63.8', 'I63.81', 'I63.89', 'I63.9', 'Z86.73', 'G45.0', 'G45.1', 'G45.2', 'G45.3', 'G45.4', 'G45.8', 'G45.9', 'Z86.73'
Systolic Heart Failure	'I50.20', 'I50.21', 'I50.22', 'I50.23', 'I50.40', 'I50.41', 'I50.42', 'I50.43'
Syncope	'R55'
TR	Presence and severity extracted from echocardiographic procedure reports using validated NLP

Aortic valve treatment with AVR	‘02RF07Z’, '02RF08Z', '02RF0JZ', ‘02RF0KZ’, ‘02RF3JH’, ‘02RF3KH’, ‘02RF37H’, ‘02RF38H’, ‘02RF37Z’, '02RF38Z', '02RF3JZ', ‘02RF3KZ’, ‘02RF47Z’, ‘02RF48Z’, ‘02RF4JZ’, ‘02RF4KZ’, '02RX0JZ', ‘02UF0JZ’, ‘X2RF032’, ‘X2RF432’, '33361', '33362', '33363’, ‘33364’, ‘33365’, ‘33366’, ‘33367’, ‘33368’, ‘33369’, '33405', ‘33406’, '33410', '33411', ‘33412’, ‘33413’, '33863', ‘33440’
Treatment with O_2	'Z99.81'
Ventricular Fibrillation	'I49.01'

AR = aortic regurgitation; AS = aortic stenosis; AVR = aortic valve replacement; CABG = coronary artery bypass graft; CAD = coronary artery disease; COPD = chronic obstructive pulmonary disease; EMR = electronic medical record; HFrEF = heart failure with reduced ejection fraction; LVEF = left ventricular ejection fraction; MI = myocardial infarction; MR = mitral regurgitation; MS = mitral stenosis; NLP = natural language processing; PCI = percutaneous coronary intervention; TR = tricuspid regurgitation.

Supplemental Table 2. Performance of Natural Language Processing-based Algorithm to Identify Diagnosed Valvular Heart Disease Severity*

	Metric, % (95% CI)		
Category	Within-label accuracy	Sensitivity	Specificity
No diagnosis found	100.00 (99.62-100.00)	100.00 (99.62-100.00)	100.00 (99.95-100.00)
Indeterminate severity	99.20 (98.43-99.59)	100.00 (99.61-100.0)	99.89 (99.78-99.94)
None	100.00 (99.62-100.00)	99.50 (98.84-99.79)	100.00 (99.95-100.00)
Mild	100.00 (99.62-100.00)	99.90 (99.44-99.98)	100.00 (99.95-100.00)
Mild-to-moderate	100.00 (99.62-100.00)	100.00 (99.62-100.00)	100.00 (99.95-100.00)
Moderate	99.90 (99.44-99.98)	99.90 (99.44-99.98)	99.99 (99.93-100.00)
Moderate-to-severe	100.00 (99.62-100.00)	99.90 (99.44-99.98)	100.00 (99.95-100.00)
Severe	99.90 (99.44-99.98)	99.80 (99.27-99.95)	99.99 (99.93-100.00)
Overall	99.88 (99.78-99.94)	99.88 (99.78-99.94)	99.98 (99.96-99.99)

*As assessed via random deidentified sampling exercise of n = 1,000 echocardiographic reports per labeling category (n = 8,000 reports total).

Supplemental Figure 1A. Two-Year Mortality Per Clinical Presentation

Sensitivity Analysis with 12-month Before-AVR Window

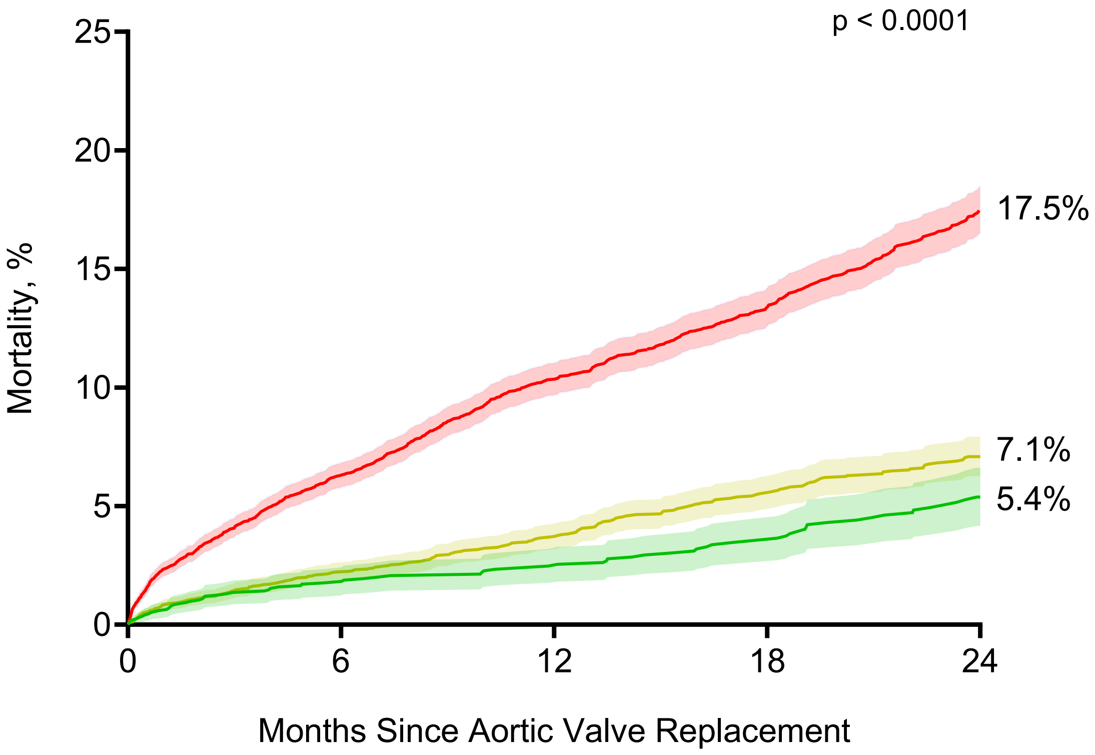

Mortality for patients at two years post AVR index 5.4% (4.1%-6.6%), 7.1% (6.2%-7.9%), 17.5% (16.5%-18.5%) with asymptomatic, progressive valve syndrome, acute valve syndrome presentation respectively.

Supplemental Figure 1B. Two-Year Hospitalization with Heart Failure Per Clinical Presentation; Sensitivity Analysis with 12-month Before-AVR Window

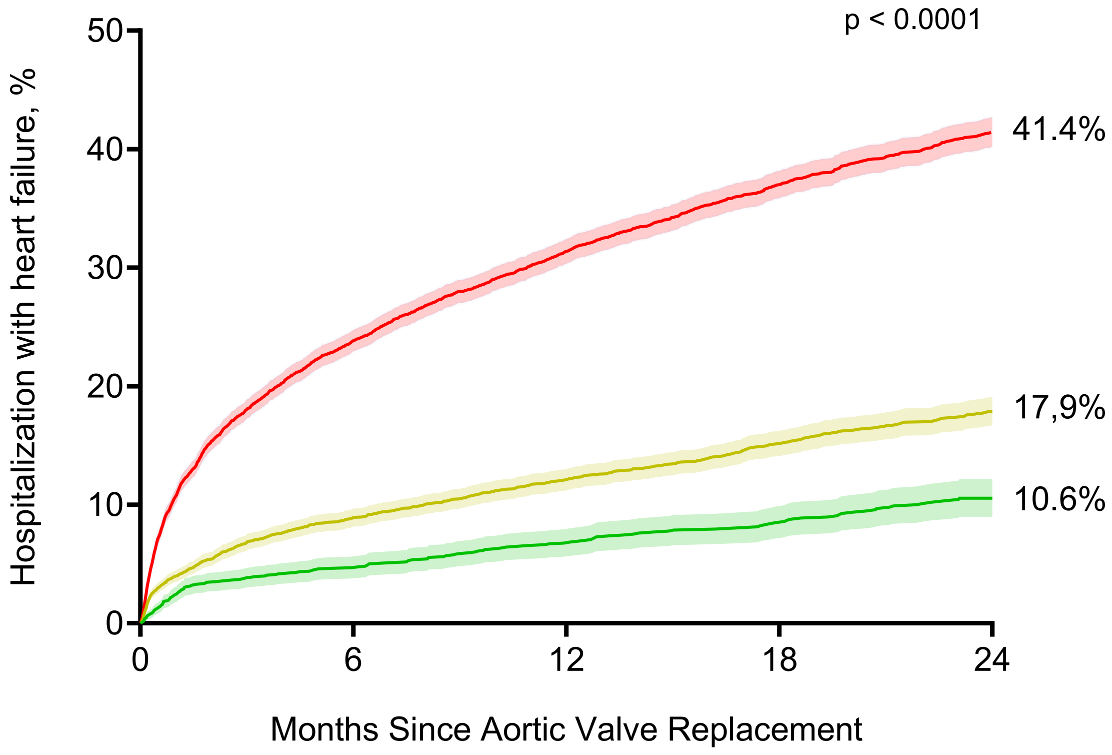

Hospitalization with heart failure at two years post AVR index 10.6% (8.9%-12.1%), 17.9% (16.7%-19.1%), 41.4% (40.1%-42.7%) with asymptomatic, progressive valve syndrome, acute valve syndrome presentation respectively.

Supplemental Figure 1C. Two-Year Mortality or Hospitalization with Heart Failure Per Clinical Presentation; Sensitivity Analysis with 12-month Before-AVR Window

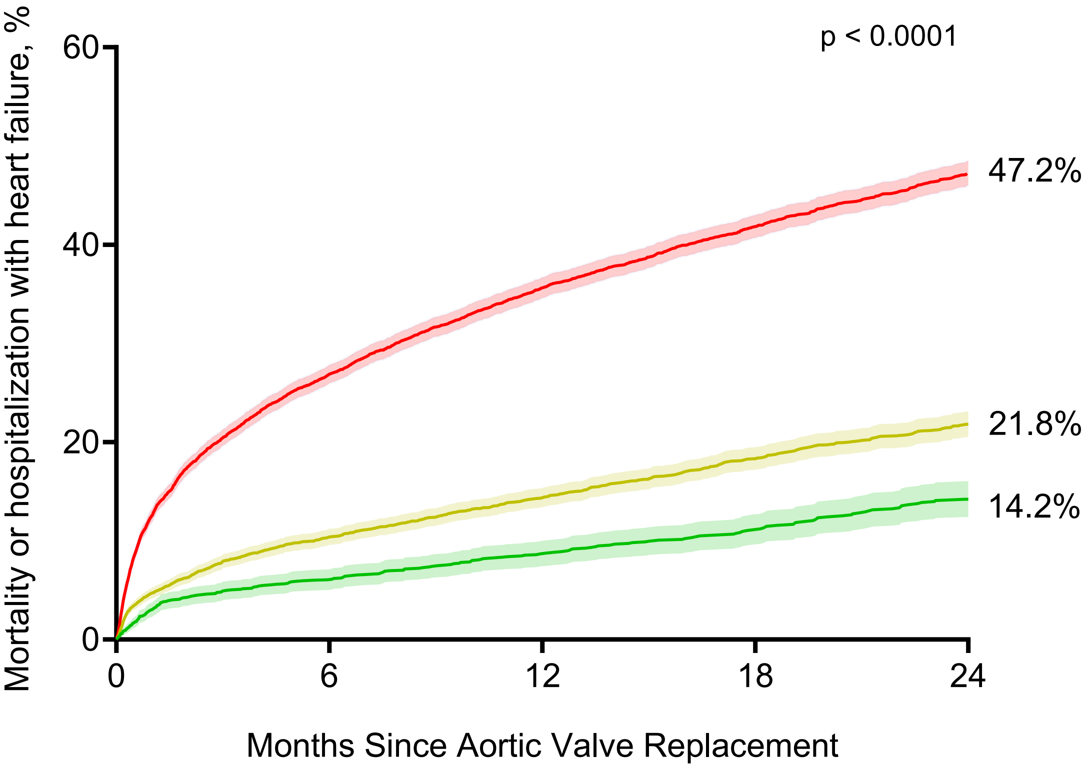

The composite of mortality and hospitalization with heart failure at two years post AVR index 14.2% (12.4%-16.0%), 21.8% (20.5%-23.1%), 47.2% (46.0%-48.5%) with asymptomatic, progressive valve syndrome, acute valve syndrome presentation respectively.

Supplemental Figure 2A. Multiple Variable Analysis for Two-Year Mortality After Aortic Valve Replacement; Sensitivity Analysis with 12-month Before-AVR Window

Adjusted for age, sex, comorbidities, and clinical presentation before aortic valve replacement.

Covariate	HR (95% CI)
Acute Valve Syndrome	2.2 (1.9 to 2.7)
Progressive Valve Syndrome	1.1 (0.9 to 1.3)
Age (Decade)	1.5 (1.5 to 1.6)
Male	1.1 (1.0 to 1.2)
COPD	1.5 (1.4 to 1.7)
Diabetes	1.3 (1.2 to 1.4)
Myocardial Infarction	1.3 (1.2 to 1.5)
Renal Disease	1.7 (1.6 to 1.9)
Stroke	1.2 (1.0 to 1.3)

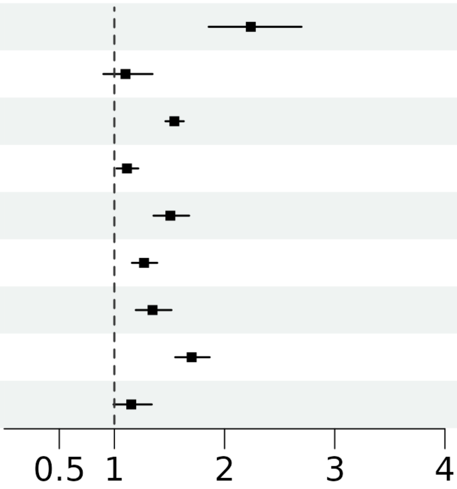

Supplemental Figure 2B. Multiple Variable Analysis for Two-Year Hospitalization with Heart Failure After Aortic Valve Replacement; Sensitivity Analysis with 12-month Before-AVR Window; Adjusted for age, sex, comorbidities, and clinical presentation before aortic valve replacement.

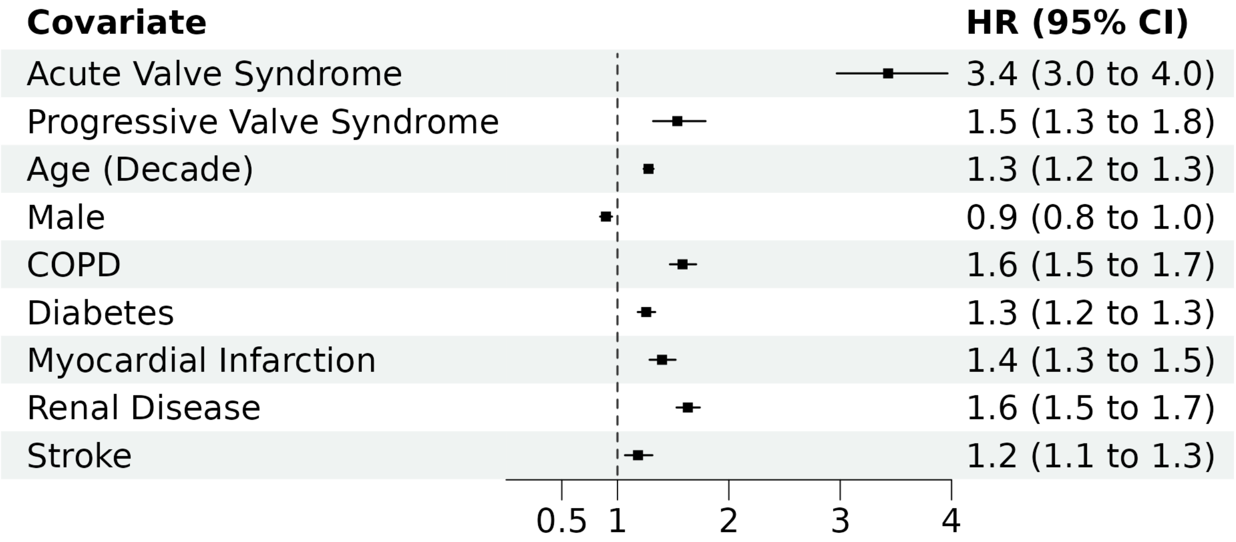

Supplemental Figure 2C. Multiple Variable Analysis for Two-Year Mortality or Hospitalization with Heart Failure After Aortic Valve Replacement; Sensitivity Analysis with 12-month Before-AVR Window; Adjusted for age, sex, comorbidities, and clinical presentation before aortic valve replacement.

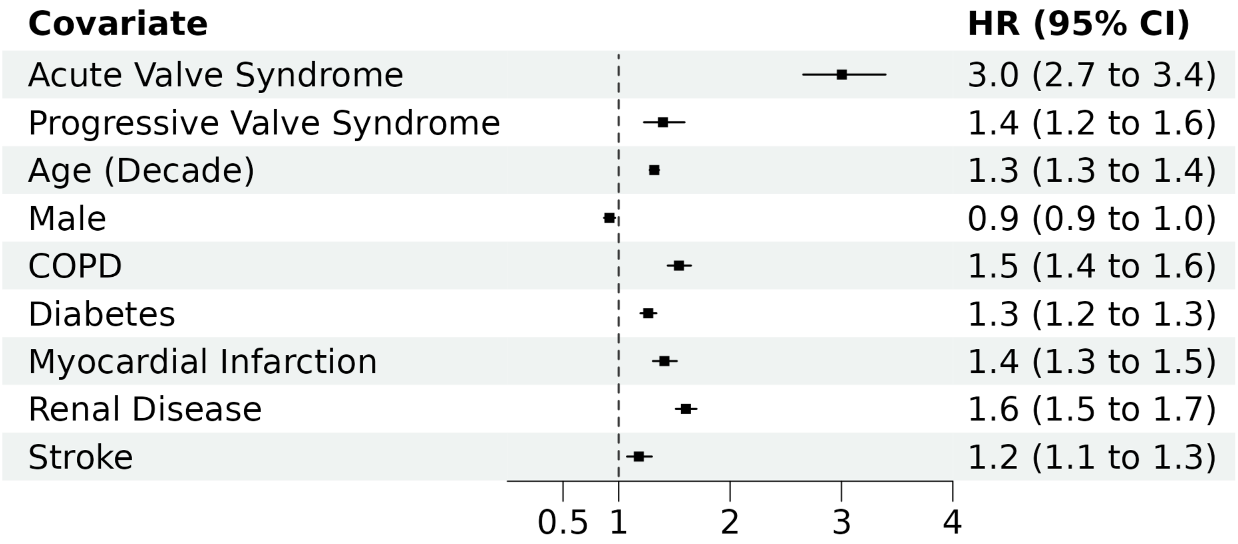

Supplementary Table 2

Stratification of Patients Undergoing AVR per 4 Groups of Clinical Presentation

**No Signs or Symptoms -- n (%)**	**2504 (14%)**
**Progressive Valve Syndrome -- n (%)**	**6116 (34.3%)**
NYHA II (Any of Dyspnea, Fatigue, Dizziness, Angina)	4575 (74.8%)
Dyspnea	3999 (65.4%)
Diagnosed diastolic heart failure	2997 (49%)
Fatigue	870 (14.2%)
Edema	630 (10.3%)
Dizziness	529 (8.6%)
Elevated natriuretic peptide, defined as 100 < BNP < 400 pg/mL and/or 1,000 pg/mL < NT-proBNP <1,500 pg/mL	488 (8%)
Angina	446 (7.3%)
**Advanced Valve Syndrome -- n (%)**	**5508 (30.9%)**
Admission with heart failure	2789 (50.6%)
LV ejection fraction <50% (echocardiography)	2200 (39.9%)
Systolic HF	1985 (36%)
High natriuretic peptide, defined as BNP ≥400 pg/mL and/or NT-proBNP ≥1,500 pg/mL	1469 (26.7%)
New-onset atrial fibrillation	1288 (23.4%)
**Acute Valve Syndrome -- n (%)**	**3710 (20.8%)**
Syncope	1494 (40.3%)
Hypotension	1295 (34.9%)
Pulmonary Edema	1049 (28.3%)
Cardiogenic Shock	357 (9.6%)
New-onset ventricular arrythmia	329 (8.9%)
Endocarditis	76 (2%)
Resuscitation from cardiac arrest	39 (1.1%)

Values are n (%)

Supplementary Figure 3A

Two-year mortality after AVR per clinical presentation before AVR.

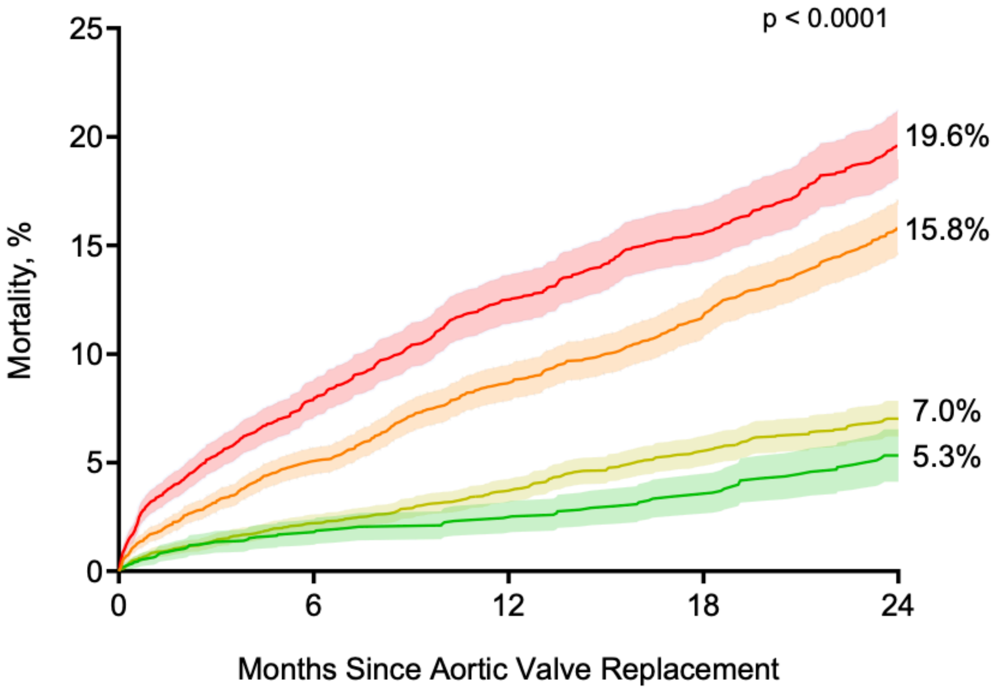

No. at Risk

	0	6	12	18	24
Asymptomatic	2250	1667	1387	1065	852
Progressive	5953	4577	3764	2793	2087
Advanced	5545	4068	3241	2306	1682
Acute	4090	2924	2258	1585	1160

Supplementary Figure 3B

Two-year hospitalization with heart failure after AVR per clinical presentation before AVR.

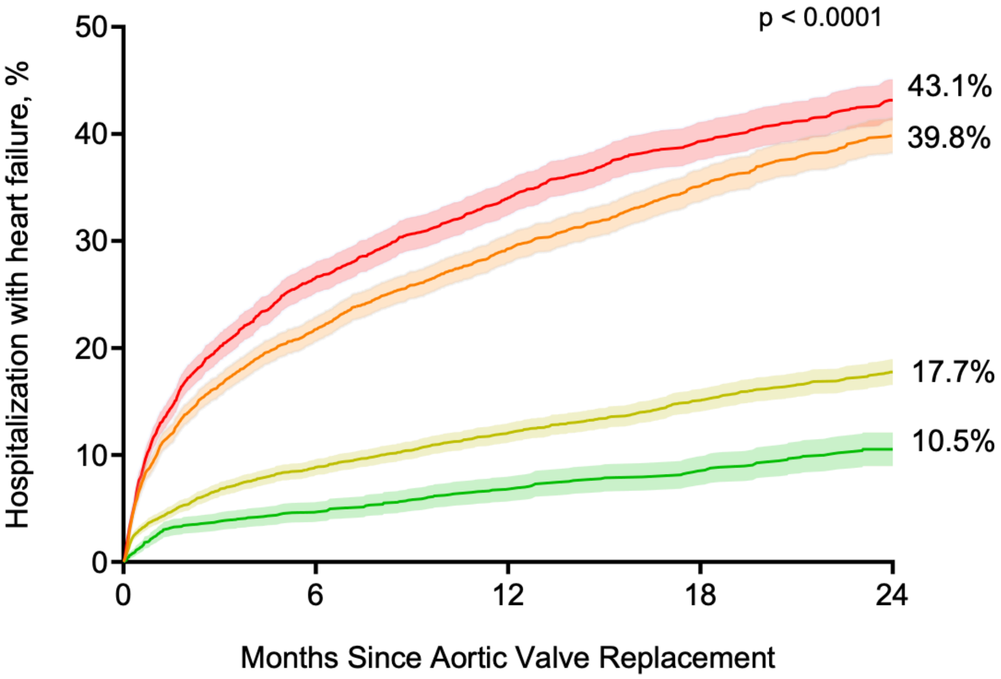

No. at Risk					
Asymptomatic	2250	1598	1315	998	785
Progressive	5953	4216	3365	2428	1762
Advanced	5545	3261	2397	1592	1093
Acute	4090	2220	1599	1061	757

Supplementary Figure 3C

Two-year mortality or hospitalization with heart failure after AVR per clinical presentation before AVR.

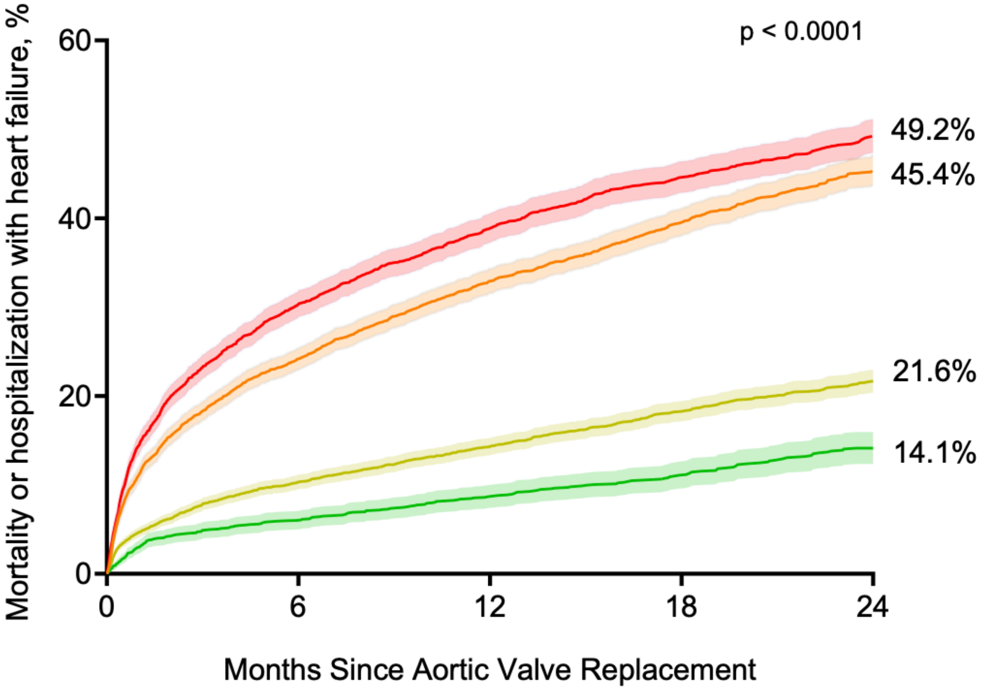

No. at Risk					
Asymptomatic	2250	1598	1315	998	785
Progressive	5953	4216	3365	2428	1762
Advanced	5545	3261	2397	1592	1093
Acute	4090	2220	1599	1061	757

Supplementary Figure 3D

Multiple Variable Analysis for Two-Year Mortality After Aortic Valve Replacement
Adjusted for age, sex, comorbidities, and clinical presentation before aortic valve replacement.

Covariate	HR (95% CI)
Acute Valve Syndrome	2.4 (2.0 to 3.0)
Advanced Valve Syndrome	2.1 (1.8 to 2.6)
Progressive Valve Syndrome	1.1 (0.9 to 1.4)
Age (Decade)	1.5 (1.5 to 1.6)
Male	1.1 (1.0 to 1.2)
COPD	1.5 (1.3 to 1.7)
Diabetes	1.3 (1.2 to 1.4)
Myocardial Infarction	1.3 (1.2 to 1.5)
Renal Disease	1.7 (1.6 to 1.9)
Stroke	1.1 (1.0 to 1.3)

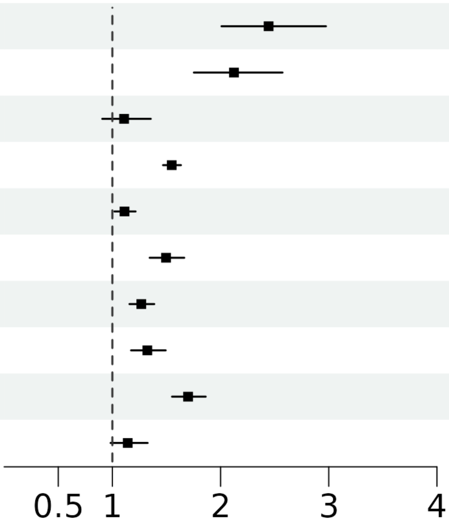

Supplementary Figure 3E

Multiple Variable Analysis for Two-Year Hospitalization with Heart Failure After Aortic Valve Replacement
Adjusted for age, sex, comorbidities, and clinical presentation before aortic valve replacement.

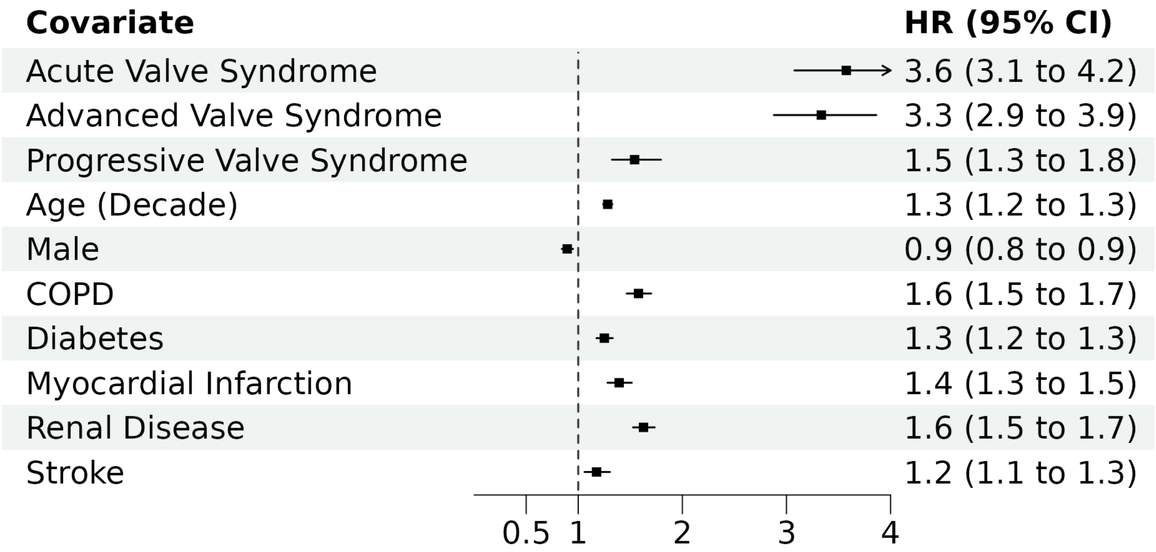

Supplementary Figure 3F

Multiple Variable Analysis for Two-Year Composite of mortality or hospitalization with heart failure After Aortic Valve Replacement

Adjusted for age, sex, comorbidities, and clinical presentation before aortic valve replacement.

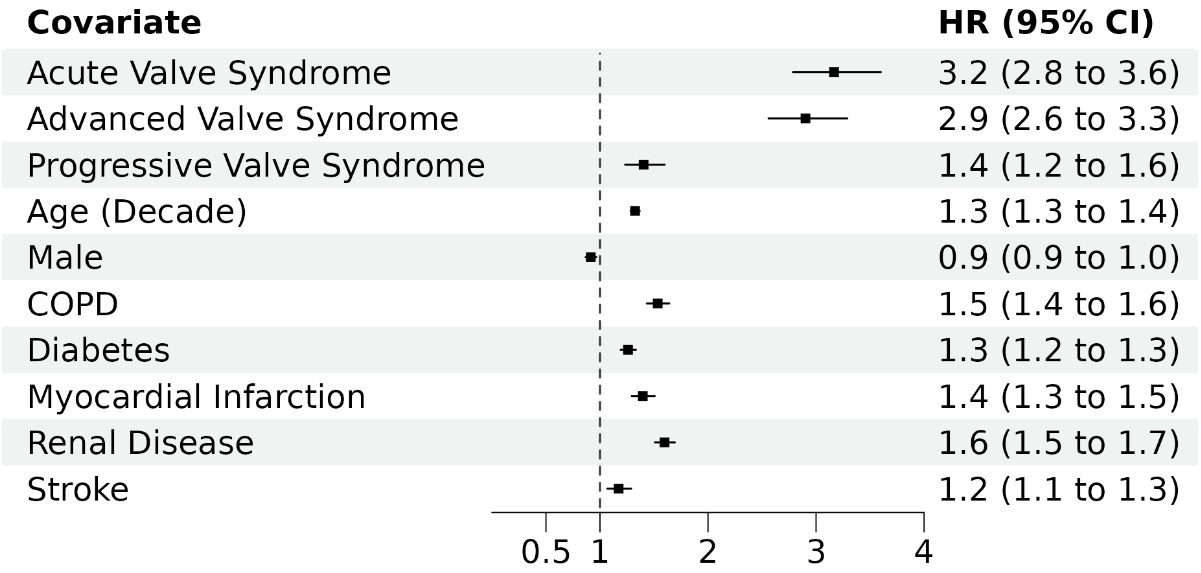

Supplement: Supplementary Appendix [file mmc1.pdf]
